# Supplementary material for: Time to Step Up Conservation: Climate Change Will Further Reduce the Suitable Habitats for the Vulnerable Species Marbled Polecat (Vormela peregusna)
Source: Animals (Basel). 2023 Jul 18;13(14):2341. doi: 10.3390/ani13142341 (PMC10376176; doi:10.3390/ani13142341)

Supplementary materials for

Time to Step up Conservation: Climate Change Will Further Reduce the Suitable Habitats for the Vulnerable Species Marbled Polecat (*Vormela peregusna*)

Xiaotian Cheng [1], Yamin Han [1], Jun Lin [2], Fan Jiang [3], Qi Cai [4], Yong Shi [3], Dongyang Cui [3] and Xuanye Wen [3,*]

1 The Station of Forest Seedling Quarantine and Pest Management, 831100 Changji, China; chengxiaotian8862@163.com (X.C.); hym0727@126.com (Y.H.)
2 Locust and Rodent Control Headquarters of Xinjiang Uygur Autonomous Region, 830000 Urumqi, China; jun_lin2013@163.com
3 Center for Biological Disaster Prevention and Control, National Forestry and Grassland Administration, 110031 Shenyang, China; jiangfan930430@163.com (F.J.); yshisyau@163.com (Y.S.); cuidy@bdpc.org.cn (D.C.)
4 Institute of Ecological Conservation and Restoration, Chinese Academy of Forestry, 100091 Beijing, China; caiqilinda1010@126.com
* Correspondence: wenxuanye_1116@163.com; Tel.: +86-186-0440-0387

Talbe S1. Distribution points of *Vormela peregusna*

Number	Species	Longitude	Latitude
1	*Vormela peregusna*	35.043	32.61
2	*Vormela peregusna*	34.941	32.516
3	*Vormela peregusna*	34.436	30.899
4	*Vormela peregusna*	35.137	32.837
5	*Vormela peregusna*	35.576	32.881
6	*Vormela peregusna*	35.57	33.207
7	*Vormela peregusna*	35.53	32.97
8	*Vormela peregusna*	34.97	32.467
9	*Vormela peregusna*	34.87	31.38
10	*Vormela peregusna*	34.92	32.53
11	*Vormela peregusna*	34.91	32.28
12	*Vormela peregusna*	34.82	31.214
13	*Vormela peregusna*	44.39222	35.46806
14	*Vormela peregusna*	28.938777	45.15925
15	*Vormela peregusna*	28.875195	45.160312
16	*Vormela peregusna*	28.811609	45.161338
17	*Vormela peregusna*	28.55357	45.030113
18	*Vormela peregusna*	28.749398	45.207317
19	*Vormela peregusna*	28.875195	45.160312
20	*Vormela peregusna*	28.487788	44.940968
21	*Vormela peregusna*	29.187908	45.019718
22	*Vormela peregusna*	28.177446	45.259656
23	*Vormela peregusna*	28.305904	45.303267
24	*Vormela peregusna*	29.187908	45.019718
25	*Vormela peregusna*	29.267361	45.423246

26	*Vormela peregusna*	28.938777	45.15925
27	*Vormela peregusna*	28.033013	44.315806
28	*Vormela peregusna*	28.472906	44.355995
29	*Vormela peregusna*	28.65348	44.083429
30	*Vormela peregusna*	28.662328	44.39841
31	*Vormela peregusna*	28.582676	43.769306
32	*Vormela peregusna*	28.546316	44.760146
33	*Vormela peregusna*	28.662328	44.39841
34	*Vormela peregusna*	28.650979	43.993431
35	*Vormela peregusna*	28.875195	45.160312
36	*Vormela peregusna*	28.811609	45.161338
37	*Vormela peregusna*	28.427784	45.076765
38	*Vormela peregusna*	28.468417	44.175991
39	*Vormela peregusna*	28.28277	44.268259
40	*Vormela peregusna*	27.716273	44.048133
41	*Vormela peregusna*	28.486627	44.895973
42	*Vormela peregusna*	28.361075	44.942549
43	*Vormela peregusna*	29.267361	45.423246
44	*Vormela peregusna*	28.215508	44.043912
45	*Vormela peregusna*	28.365349	45.122538
46	*Vormela peregusna*	28.875195	45.160312
47	*Vormela peregusna*	28.589847	44.039315
48	*Vormela peregusna*	28.539164	44.490165
49	*Vormela peregusna*	28.649732	43.948431
50	*Vormela peregusna*	28.685756	45.208275
51	*Vormela peregusna*	28.432288	45.256744
52	*Vormela peregusna*	28.875195	45.160312
53	*Vormela peregusna*	28.742542	44.982375

54	*Vormela peregusna*	28.033013	44.315806
55	*Vormela peregusna*	27.976314	44.676417
56	*Vormela peregusna*	28.177446	45.259656
57	*Vormela peregusna*	28.662328	44.39841
58	*Vormela peregusna*	27.716273	44.048133
59	*Vormela peregusna*	28.803113	44.891413
60	*Vormela peregusna*	28.177446	45.259656
61	*Vormela peregusna*	28.65348	44.083429
62	*Vormela peregusna*	28.803113	44.891413
63	*Vormela peregusna*	28.662328	44.39841
64	*Vormela peregusna*	27.655353	44.183549
65	*Vormela peregusna*	28.432288	45.256744
66	*Vormela peregusna*	28.432288	45.256744
67	*Vormela peregusna*	28.472906	44.355995
68	*Vormela peregusna*	28.242142	45.303979
69	*Vormela peregusna*	28.468417	44.175991
70	*Vormela peregusna*	28.65348	44.083429
71	*Vormela peregusna*	28.926682	44.799372
72	*Vormela peregusna*	28.177446	45.259656
73	*Vormela peregusna*	28.650979	43.993431
74	*Vormela peregusna*	28.360013	44.897551
75	*Vormela peregusna*	28.157502	44.269597
76	*Vormela peregusna*	28.65348	44.083429
77	*Vormela peregusna*	28.427784	45.076765
78	*Vormela peregusna*	28.589847	44.039315
79	*Vormela peregusna*	28.033013	44.315806
80	*Vormela peregusna*	28.28277	44.268259
81	*Vormela peregusna*	27.655353	44.183549

82	*Vormela peregusna*	28.468417	44.175991
83	*Vormela peregusna*	27.963717	43.911259
84	*Vormela peregusna*	67.723692	38.539821
85	*Vormela peregusna*	65.716667	31.616671
86	*Vormela peregusna*	34.849674	32.165619
87	*Vormela peregusna*	47.064999	34.314171
88	*Vormela peregusna*	56.933331	37.516392
89	*Vormela peregusna*	48.333302	33.505508
90	*Vormela peregusna*	96.570313	61.721541
91	*Vormela peregusna*	46.715819	43.847118
92	*Vormela peregusna*	96.570313	61.721541
93	*Vormela peregusna*	28.53	45.02
94	*Vormela peregusna*	28.43	44.37
95	*Vormela peregusna*	82.4851	45.0422
96	*Vormela peregusna*	34.591193	31.444447
97	*Vormela peregusna*	100.170554	39.158514
98	*Vormela peregusna*	87.857	44.413
99	*Vormela peregusna*	107.735588	37.809974
100	*Vormela peregusna*	85.732745	46.79415
101	*Vormela peregusna*	127.412612	46.090552

Figure S1 Comparison of AUC, kappa coefficient, and TSS evaluations of 9 models.

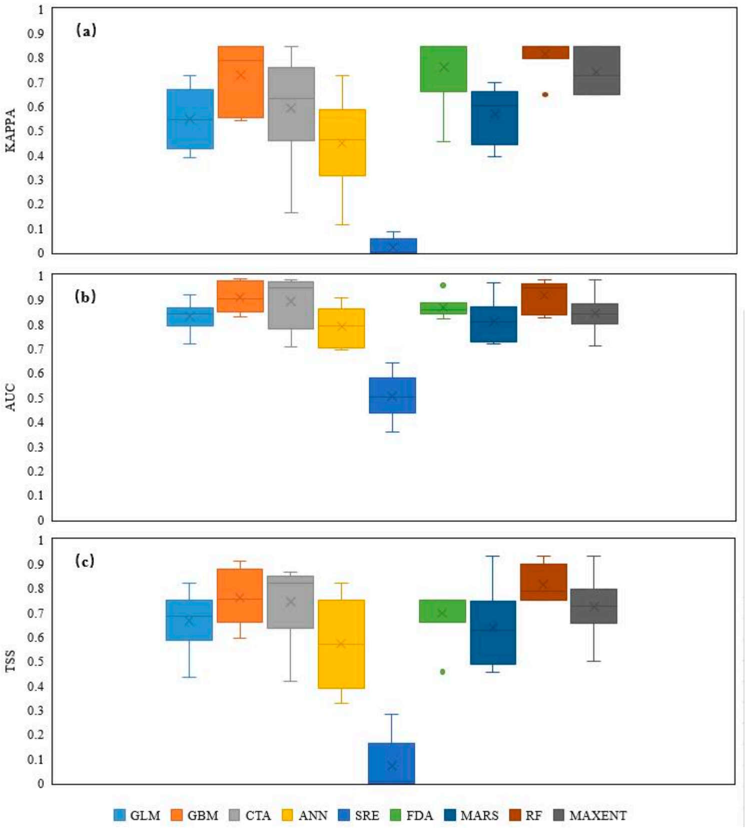

Supplement: Supplementary file 1 [file animals-13-02341-s001.zip › animals-2468882-supplementary.pdf]
